# Electronic Decision Support for Deprescribing in Patients on Hemodialysis: Clinical Research Protocol for a Prospective, Controlled, Quality Improvement Study

**DOI:** 10.1177/20543581231165712

**Published:** 2023-06-26

**Authors:** Émilie Bortolussi-Courval, Tiina Podymow, Emilie Trinh, Joseph Moryousef, R. Hanula, Jean-François Huon, Thomas Mavrakanas, Rita Suri, Todd C. Lee, Emily Gibson McDonald

**Affiliations:** 1 Division of Experimental Medicine, Department of Medicine, McGill University Health Centre, Montreal, QC, Canada; 2 Division of Nephrology, Department of Medicine, McGill University Health Centre, Montreal, QC, Canada; 3 Faculty of Medicine and Health Sciences, McGill University, Montreal, QC, Canada; 4 Department of Medicine, McGill University Health Centre, Montreal, QC, Canada; 5 Division of Infectious Diseases, Department of Medicine, McGill University Health Centre, Montreal, QC, Canada; 6 Clinical Practice Assessment Unit, Department of Medicine, McGill University Health Centre, Montreal, QC, Canada; 7 Division of General Internal Medicine, Department of Medicine, McGill University Health Centre, Montreal, QC, Canada; 8 Centre for Outcomes Research and Evaluation, Montreal, QC, Canada

**Keywords:** hemodialysis, dialysis, polypharmacy, deprescribing, end-stage kidney disease

## Abstract

**Background:**

Patients on dialysis are commonly prescribed multiple medications (polypharmacy), many of which are potentially inappropriate medications (PIMs). Potentially inappropriate medications are associated with an increased risk of falls, fractures, and hospitalization. MedSafer is an electronic tool that generates individualized, prioritized reports with deprescribing opportunities by cross-referencing patient health data and medications with guidelines for deprescribing.

**Objectives:**

Our primary aim was to increase deprescribing, as compared with usual care (medication reconciliation or MedRec), for outpatients receiving maintenance hemodialysis, through the provision of MedSafer deprescribing opportunity reports to the treating team and patient empowerment deprescribing brochures provided directly to the patients themselves.

**Design:**

This controlled, prospective, quality improvement study with a contemporary control builds on existing policy at the outpatient hemodialysis centers where biannual MedRecs are performed by the treating nephrologist and nursing team.

**Setting:**

The study takes place on 2 of the 3 outpatient hemodialysis units of the McGill University Health Centre in Montreal, Quebec, Canada. The intervention unit is the Lachine Hospital, and the control unit is the Montreal General Hospital.

**Patients:**

A closed cohort of outpatient hemodialysis patients visit one of the hemodialysis centers multiple times per week for their hemodialysis treatment. The initial cohort of the intervention unit includes 85 patients, whereas the control unit has 153 patients. Patients who are transplanted, hospitalized during their scheduled MedRec, or die before or during the MedRec will be excluded from the study.

**Measurements:**

We will compare rates of deprescribing between the control and intervention units following a single MedRec. On the intervention unit, MedRecs will be paired with MedSafer reports (the intervention), and on the control unit, MedRecs will take place without MedSafer reports (usual care). On the intervention unit, patients will also receive deprescribing patient empowerment brochures for select medication classes (gabapentinoids, proton-pump inhibitors, sedative hypnotics and opioids for chronic non-cancer pain). Physicians on the intervention unit will be interviewed post-MedRec to determine implementation barriers and facilitators.

**Methods:**

The primary outcome will be the proportion of patients with 1 or more PIMs deprescribed on the intervention unit, as compared with the control unit, following a biannual MedRec. This study will build on existing policies aimed at optimizing medication therapy in patients undergoing maintenance hemodialysis. The electronic deprescribing decision support tool, MedSafer, will be tested in a dialysis setting, where nephrologists are regularly in contact with patients. MedRecs are an interdisciplinary clinical activity performed biannually on the hemodialysis units (in the Spring and Fall), and within 1 week following discharge from any hospitalization. This study will take place in the Fall of 2022. Semi-structured interviews will be conducted among physicians on the intervention unit to determine barriers and facilitators to implementation of the MedSafer-supplemented MedRec process and analyzed according to grounded theory in qualitative research.

**Limitations:**

Deprescribing can be limited due to nephrologists’ time constraints, cognitive impairment of the hemodialyzed patient stemming from their illness and complex medication regimens, and lack of sufficient patient resources to learn about the medications they are taking and their potential harms.

**Conclusions:**

Electronic decision support can facilitate deprescribing for the clinical team by providing a nudge reminder, decreasing the time it takes to review and effectuate guideline recommendations, and by lowering the barrier of when and how to taper. Guidelines for deprescribing in the dialysis population have recently been published and incorporated into the MedSafer software. To our knowledge, this will be the first study to examine the efficacy of pairing these guidelines with MedRecs by leveraging electronic decision support in the outpatient dialysis population.

**Trial registration:**

This study was registered on Clinicaltrials.gov (NCT05585268) on October 2, 2022, prior to the enrolment of the first participant on October 3, 2022. The registration number is pending at the time of protocol submission.

## Abrégé

**Contexte:**

Les patients sous dialyse se voient souvent prescrire de nombreux médicaments (polypharmacie), dont plusieurs médicaments potentiellement inappropriés (MPI). Les MPI sont associés à un risque accru de chutes, de fractures et d’hospitalisations. MedSécure est un outil électronique qui génère des rapports individualisés et classés par ordre de priorité indiquant les possibilités de déprescription. L’outil fonctionne en croisant les données sur la santé des patients et les médicaments sous ordonnance avec des lignes directrices pour la déprescription.

**Objectifs de l’étude:**

L’objectif principal est de favoriser la déprescription par rapport aux soins habituels (*Medication Reconciliation* [MedRecs] ou bilan comparatif des médicaments) chez les patients ambulatoires recevant une hémodialyse d’entretien, en fournissant des rapports MedSécure de déprescription à l’équipe soignante et des brochures encourageant la déprescription aux patients.

**Conception:**

Cette étude prospective et contrôlée (témoin contemporain) d’amélioration de la qualité s’appuie sur la politique existante dans les centers d’hémodialyse ambulatoires où un bilan des médicaments (MedRecs) est effectué deux fois par année par le néphrologue traitant et l’équipe de soins infirmiers.

**Cadre:**

L’étude a lieu dans deux des trois unités d’hémodialyse ambulatoire du Center universitaire de santé McGill à Montréal (Québec, Canada). L’unité d’intervention est l’Hôpital de Lachine et l’unité témoin est l’Hôpital général de Montréal.

**Sujets:**

Une cohorte fermée de patients ambulatoires sous hémodialyse qui visitent plusieurs fois par semaine un center d’hémodialyse pour leurs traitements. La cohorte initiale de l’unité d’intervention compte 85 patients, tandis que l’unité témoin compte 132 patients. Seront exclus les patients qui recevront une greffe, qui seront hospitalisés pendant leur MedRecs ou qui décèderont avant ou pendant le MedRecs.

**Mesures:**

Nous comparerons les taux de déprescription entre les unités témoin et d’intervention après un seul MedRecs. Dans l’unité d’intervention, le MedRecs sera associé aux rapports MedSécure (l’intervention); dans l’unité témoin, le MedRecs aura lieu sans rapports MedSécure (soins habituels). Au sein de l’unité d’intervention, les patients recevront également des brochures encourageant la déprescription pour certaines classes de médicaments (gabapentinoïdes, inhibiteurs de la pompe à protons, hypnotiques sédatifs et opioïdes pour les douleurs chroniques non cancéreuses). Les médecins de l’unité d’intervention seront interviewés après le MedRec pour déterminer les obstacles et les facilitateurs à la mise en œuvre.

**Méthodologie:**

Le principal critère d’évaluation sera la proportion de patients dans l’unité d’intervention chez qui au moins un MPI sera déprescrit après un MedRec semestriel, par rapport à l’unité témoin. L’étude s’appuiera sur les politiques existantes visant à optimiser la médication chez les patients suivant des traitements d’hémodialyse d’entretien. L’outil électronique d’aide à la décision de déprescription MedSécure sera testé en contexte de dialyse, où les néphrologues sont régulièrement en contact avec les patients. Les MedRecs sont une activité clinique interdisciplinaire effectuée semestriellement sur les unités d’hémodialyse (au printemps et à l’automne) et dans la semaine suivant un congé de l’hôpital. Cette étude aura lieu à l’automne 2022. Des entretiens semi-structurés seront menés avec les médecins de l’unité d’intervention afin d’établir les obstacles et les facilitateurs à la mise en œuvre du processus MedRec complété par MedSécure, puis analysés selon une théorie fondée sur la recherche qualitative.

**Limites:**

La déprescription peut être limitée par des contraintes de temps des néphrologues, des troubles cognitifs résultant des maladies et des régimes médicamenteux complexes des patients sous hémodialyse ou par un manque de ressources pour éduquer les patients sur les médicaments qu’ils prennent et leurs méfaits potentiels.

**Conclusion:**

Un outil électronique d’aide à la décision peut faciliter le processus de déprescription pour l’équipe clinique en fournissant un rappel, en réduisant le temps nécessaire à l’examen et à l’application des recommandations, et en limitant les obstacles liés au moment et à la façon de réduire le nombre de médicaments. Des lignes directrices sur la déprescription dans la population des patients sous dialyse ont récemment été publiées et incorporées au logiciel MedSécure. À notre connaissance, il s’agit de la première étude à examiner l’efficacité du couplage des lignes directrices avec le MedRecs en tirant parti de l’outil électronique d’aide à la décision en contexte d’hémodialyse ambulatoire.

## Introduction

### Background and Rationale

Patients with end-stage kidney disease (ESKD) who require dialysis are prescribed an average of 10 to 12 daily medications, often by 4 to 5 different clinicians, amounting to as many as 19 pills per day.^1-4^ The dialysis patient population has one of the heaviest pill burdens of all chronic conditions due to concurrent treatment of co-existing conditions such as hypertension, vascular disease, and diabetes,^4^ as well as treatment of complications of ESKD itself (eg, increased propensity for bleeding, bone mineral metabolism disorders, pruritus, pain, and insomnia).

More than 90% of dialysis patients take 5 or more medications (polypharmacy),^2,5^ contributing to medication overload.^6,7^ Furthermore, more than 90% are prescribed 1 or more potentially inappropriate medications (PIMs)^1,4,8^; PIMs are associated with an increased risk of harm (often through adverse drug events, ADEs) or they have a relatively low chance for benefit and merely contribute to a patient’s pill burden.^2,4,7^ Adverse drug events such as falls, fractures, and cognitive impairment can occur as a direct result of PIMs, or due to drug-drug interactions, both of which are more common with polypharmacy.^9,10^ Polypharmacy and ADEs contribute to emergency room visits, hospital admissions, loss of autonomy, and premature death.^11-13^ There is, therefore, a pressing need for pragmatic, scalable interventions to address polypharmacy in dialysis patients.^3,14,15^

We previously demonstrated that MedSafer, an electronic decision support tool, can increase deprescribing for hospitalized older adults and for people residing in long-term care homes.^16,17^ MedSafer identifies deprescribing opportunities by electronically cross-referencing a person’s usual medication list and medical comorbidities, with a curated ruleset containing evidence-based deprescribing guidelines (based on criteria from the American Geriatrics Society,^18^ STOPP,^19^ and Choosing Wisely^20^).^17^ MedSafer stratifies deprescribing opportunities into high-risk, intermediate-risk, or little added value categories. High risk implies there is an elevated risk of developing ADEs; intermediate risk implies that the harms must be weighed against the benefits of the drug; and drugs of little added value simply increase the pill burden of a patient or have evidence demonstrating no effect. Examples of a typical deprescribing report and of different drugs that fall into the 3 categories can be found in the Supplement of the MedSafer Study.^16^ Our large cluster randomized clinical trial of older adults hospitalized in the acute care setting (N = 5698) included 140 patients receiving maintenance hemodialysis. The net deprescribing rate among dialysis patients was 9.4% (95% confidence interval [CI] = 1.3%-17.6%) higher in the intervention period compared with the control period. While effective, the general population in the study benefited from a much higher rate of deprescribing (absolute increase of 22.2%, 95% CI = 16.9%-27.4%).^16^ At that time, no dialysis-specific rules were integrated into the MedSafer software, and patients were enrolled during a time of acute illness.

Since then, McIntyre et al^15^ have developed deprescribing algorithms specifically for hemodialysis patients in a quality improvement study.^4,8^ However, like most guidelines, there are barriers to implementation and uptake. Guidelines for deprescribing contain long lists of rules that preclude memorization; some may contain conflicting recommendations for patients taking 10 to 15 medications; and not all guidelines explain how to deprescribe (eg, when and how to taper and what rebound symptoms to watch out for).^2^,^21-23^ We therefore integrated the dialysis-specific deprescribing recommendations into the MedSafer ruleset, coupled them with links to patient deprescribing empowerment brochures, and added instructions for tapering where required.

### Objectives

Our primary aim in this study is to increase deprescribing, as compared with usual care (medication reconciliation or MedRec), for outpatients receiving maintenance hemodialysis, through the provision of MedSafer deprescribing opportunity reports to the treating team and patient empowerment deprescribing brochures provided directly to the patients.

### Study Design

This is a controlled, prospective, quality improvement study, with a contemporary control. Publication of the study will follow the SQUIRE 2.0 reporting guideline: Revised Standard for Quality Improvement Reporting Standards.^24^ Relevant elements of SQUIRE 2.0 have been addressed in this protocol (Supplemental Appendix). This protocol follows the SPIRIT^25^ checklist (Standard Protocol Items: Recommendations for Interventional Trials) and is ordered according to the *Trials* Journal Structured Study Protocol Template.^26^

## Methods: Participants, Intervention, and Outcomes

### Study Setting

The study takes place on the 2 largest (of 3) outpatient hemodialysis units at the McGill University Health Centre in Montreal, Quebec, Canada. The intervention unit is the Lachine Hospital dialysis unit, and the control unit is the Montreal General Hospital dialysis unit. This assignment was random.

### Eligibility Criteria

#### Inclusion criteria

Outpatients receiving maintenance hemodialysis on one of the two participating units.
Aged 18 years and over.
One or more PIMs identified.

#### Exclusion criteria

Patient is currently hospitalized or is planned to undergo a transplant during the time of the MedRec.
New patient initiating maintenance hemodialysis.

### Who Will Take Informed Consent?

Not applicable (a waiver of consent was obtained for this quality improvement project; medication reviews are considered best practice in our institution for patients on dialysis).^1^

### Additional Consent Provisions for Collection and Use of Participant Data and Biological Specimens

No biological specimens will be collected as part of this trial.

### Interventions

#### Explanation of the choice of comparators

We are comparing the intervention on 2 of 3 outpatient hemodialysis units within the same hospital center. Although there are some differences between the patients on each unit, they are similar. The third unit is smaller and treats more medically complex patients, including those with active cancer and solid organ transplant. We selected our control and intervention units as they resemble typical community dialysis units to increase the external validity of our findings.

#### Intervention description

This study will build on existing policies aimed at optimizing medication therapy in patients undergoing maintenance hemodialysis. The electronic deprescribing decision support tool, MedSafer, now includes dialysis-specific deprescribing indications. For example, if a dialysis patient is receiving aspirin for primary prevention, MedSafer will generate a dialysis-specific deprescribing opportunity for the clinician stating the following: “The use of aspirin in dialysis patients increases the risk of bleeding. Also, there is little benefit of using aspirin in dialysis patients for primary prevention.” Patients who are receiving aspirin for secondary prevention will have this rule suppressed. In this way, deprescribing reports are individualized. The aspirin deprescribing opportunity is an example of a high-risk alert; a flag where the harms outweigh the benefits in most patients. Other categories include intermediate risk (harms and benefits need to be balanced and assessed by the clinician) and medications of little added value (no demonstrated value or evidence of no effect). Where relevant, tapering schedules are provided if the drug must be gradually discontinued and opportunities are linked to patient deprescribing empowerment brochures from the Canadian Medication Appropriateness and Deprescribing Network.^27,28^

The use of MedSafer reports during MedRecs will be tested in the dialysis setting, where nephrologists are regularly in contact with patients. MedRecs are an interdisciplinary clinical activity performed biannually on the hemodialysis units (in the Spring and Fall), and within 1 week following discharge from any hospitalization that occurs. This study will take place in the Fall of 2022.

The usual MedRec process occurs as follows: a dialysis nurse first validates with the patient their list of usual home medications and correlates this with the medication list provided by the pharmacy. Discrepancies between the patient’s medication list and the pharmacy’s medication list are resolved through discussion with the patient and pharmacy. Afterward, the treating nephrologist and the nurse review the patient’s list of medications and perform any necessary adjustments. The process is meant to avoid medication duplication, ensure appropriate dosing in the dialysis context, and avoid omissions. Deprescribing does not routinely take place as part of the MedRec process despite this being an opportune time to re-evaluate the ongoing necessity, harms, and benefits of PIMs. Currently, whether deprescribing occurs is nephrologist dependent.

Next, for the purpose of the study, the study lead (É.B.-C.) will enter the best medication history (described above), medications, and select laboratory values (hemoglobin A1c and serum creatinine) into MedSafer and generate reports for all patients in the study. The intervention unit (Lachine Hospital) will perform one of their usual biannual MedRecs paired with MedSafer deprescribing reports, including dialysis-specific deprescribing opportunities (the intervention). Reports will be pre-generated and provided to the interdisciplinary team, along with patient deprescribing empowerment brochures.

One of the deprescribing brochures will be a fact sheet on the topic of deprescribing, and others are given for select classes of medications (sedative-hypnotic drugs,^29^ gabapentinoids,^30^ proton-pump inhibitors [PPIs],^31^ and opioids for chronic non-cancer pain^32^). The control hemodialysis unit (Montreal General Hospital) will, concurrently, perform their usual biannual MedRec, in the absence of deprescribing reports or patient empowerment brochures.

MedSafer contains deprescribing opportunities from several existing guidelines for safer prescribing in older adults.^18,19,33^ As previously mentioned, the reports will be printed and placed in a study binder at the physician’s desk on the intervention unit and organized alphabetically according to the first letter of the last name of each patient. If MedSafer does not emit any recommendations, a report stating “no deprescribing opportunities were identified at this time” will be generated and placed in the binder.

Physicians on the intervention unit will receive a university-affiliated email from the study lead 2 weeks before the first planned start of the study to explain the workflow of the MedSafer MedRec process. They will be provided with a checklist to follow while they are performing MedRecs with the MedSafer reports as a guide. Study contact information is also provided for any support with the project. This guide (Supplemental Appendix) will ensure standardization of the MedRec process on the intervention unit. This initial email will also contain an example of a MedSafer deprescribing report. The treating physicians on the intervention unit will meet the study lead at the intervention site in person the day before the planned start of the study to review the MedSafer MedRec workflow and introduce them to the MedSafer reports and deprescribing brochures.

The patient deprescribing empowerment brochures (available in French and English) will be made available to the physician in the study binder, paired with the deprescribing reports. At the physician’s discretion, these will be provided to the patient to increase the motivation to deprescribe and provide an active learning opportunity for patients regarding their pharmacotherapy. Each patient on the intervention unit will receive a deprescribing fact sheet available in a third-grade-level language (Supplemental Appendix).

Ultimately, the decision of whether to deprescribe is left to the clinical reasoning of the treating team, and shared decision making with the patient will be encouraged, as this is a pragmatic intervention to study the real-world efficacy of making the deprescribing reports and brochures available in hemodialysis units.

#### Criteria for discontinuing or modifying allocated interventions

There are no planned interim analyses, as this is a quality improvement intervention that is considered best practice. Medication reconciliation for patients receiving maintenance hemodialysis occurs as part of usual care.

#### Strategies to improve adherence to interventions

Efforts will be made in this study to facilitate the ease of access to MedSafer reports and to iteratively improve the MedRec workflow through weekly “plan, do, study, act” cycles. To begin with, an introductory email will be sent to the nephrologists attending on the intervention unit. This email will contain an overview of the study, how it integrates with the existing workflow, and the expectations for the treating nephrologist (to review the MedSafer report at the time of performing a MedRec, perform any relevant deprescribing, provide the deprescribing brochures to the patient, and make a note in the electronic when the MedRec is complete). The study lead will generate sample MedSafer reports for the clinical team, so they can familiarize themselves with the report format prior to the intervention commencing. A physician nephrologist champion (T.P.) will be available to answer any questions or concerns.

### Relevant Concomitant Care Permitted or Prohibited During the Trial

All usual care will be permitted, and no specific care is prohibited during the study.

### Provisions for Post-trial Care

Patients in the study will continue to receive usual (usually thrice weekly) care in the hemodialysis unit, post-intervention.

### Outcomes

The *primary outcome* will be the efficacy of MedSafer for deprescribing, based on the proportion of patients with 1 or more PIMs deprescribed, compared between the intervention and control units. This will be conditioned on patients with 1 or more PIMs at baseline. Deprescribing will be defined as any PIM that is identified by the MedSafer deprescribing algorithms, that is: stopped or deliberately reduced or tapered.

The key *secondary outcomes* will be the reduction in the mean number of prescribed drugs from baseline following a MedRec compared between the intervention and control units, and implementation barriers and facilitators collected from qualitative, semi-structured interviews with nephrologists on the intervention unit. The number of gastrointestinal bleeds will be reported in total and by intervention status.

### Participant Timeline

See Figure 1.

**Figure 1.**
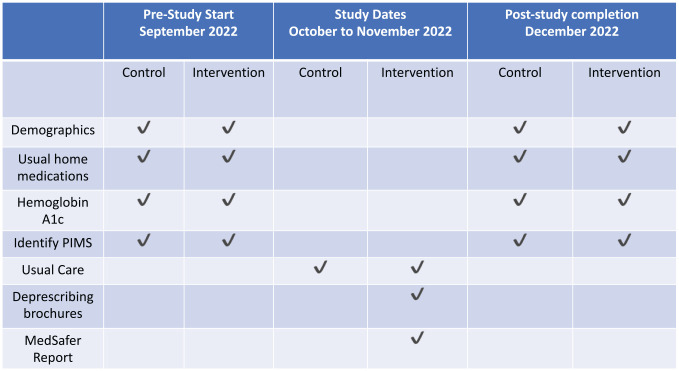
Participant timeline during the study.

### Sample Size

The sample size is fixed to the number of patients in each of the dialysis units (85 patients on the intervention unit and 153 on the control unit). A previous study of MedSafer in hemodialysis estimated ~90% of dialysis patients are prescribed 1 or more PIMs, which is the assumption we will make here as well (N = 214). In our acute care study, we found a baseline rate of PIM deprescribing in the hemodialysis subpopulation of 19.3%. However, in outpatients, deprescribing of sedative hypnotics is often as low as 5%.^27^ Therefore, we will estimate a 10% baseline rate of deprescribing. With a 2-sided alpha of 0.05, 80% power, and approximately 1:2 allocation between intervention and control, we can show an increase of at least 15% in the proportion of patients with 1 or more PIMs deprescribed. The statistical code for this calculation is available in the Supplemental Appendix.

### Recruitment

A waiver of consent was granted by the McGill University Health Centre Director of Professional Services for this Quality Improvement Intervention. For the purposes of analysis, only the initial closed cohort will be included in the study. New patients who are initiated on maintenance hemodialysis during the study will not be included. Patients who die or are transplanted prior to a MedRec, or who are admitted to hospital and cannot have a MedRec performed, will be accounted for, described, but excluded from the final analysis.

### Assignment of Interventions: Allocation and Blinding

*Sequence generation, Concealment mechanism, Implementation of Allocation*, are all not applicable to this study.

### Who Will Be Blinded?

Clinicians at the control unit will not receive MedSafer reports but will carry out a biannual MedRec as part of usual care. Clinicians rounding on the intervention unit will be made aware of the intervention approximately 2 weeks prior to starting, to allow for familiarization of the MedSafer reports. Four nephrologists will round on the intervention unit during this time, and 3 different nephrologists will round on the control. There is no crossover between physician schedules. Therefore, there is little risk of contamination of the intervention and the control site.

The *Procedure for unblinding* is not applicable to this study.

## Data Collection and Management

### Plans for Assessment and Collection of Outcomes

Baseline demographic data, the best possible medication history data, the most recent glycated hemoglobin as a measure of diabetes control, and creatinine will be collected for both the control and intervention unit from the electronic medical record (EMR), called NephroCare. These data will be input in the MedSafer web-based portal, and deprescribing reports will be generated for patients on the intervention unit.

Following a MedRec, medication changes will be captured from NephroCare and input into the MedSafer software for both the intervention and control units, permitting an analysis comparing deprescribing that took place on the 2 units.

At the time of MedRec, nephrologists will review the medication list of the patient (obtained from the EMR), validate the list with the patient, and then deprescribe when deemed appropriate (based on the MedSafer report). Medication changes will be faxed the same day to patient’s pharmacy. During this process, if a patient advises the clinical team that they are no longer taking a prescribed drug, this medication will be removed from the patient’s medication list in the EMR. If it is discovered that a patient has already discontinued a drug at the time of performing a MedRec, this will not be counted in the primary outcome of deprescribing.

At the end of the intervention, nephrologists who participated in MedRecs will be invited to participate in semi-structured interviews with the study lead to address their perceived facilitators and barriers related to deprescribing on the dialysis unit. Afterward, themes will be developed from the data collection, according to the grounded theory in qualitative research.^34^

### Plans to Promote Participant Retention and Complete Follow-up

We do not expect significant loss to follow-up given the intensity that patients on outpatient hemodialysis are monitored and the short timeframe of the intervention.

### Data Management

Manual baseline data entry for all patients will occur in the MedSafer web-based portal with data extracted from the NephroCare EMR by a trained study investigator. Any medication changes that occur are input into NephroCare at the time of performing the MedRec. When a MedRec occurs, the date is noted in the unit “task binder” located on site and accessible to all clinicians. MedRecs are considered up to date if they have been completed in the past 6 months and are only redone in the event a patient is hospitalized.

Before and after the MedRecs have been completed, each patient’s medication and comorbidity data will be updated in MedSafer, and a pre- and post-intervention medication dataset (coded by study ID) will be extracted and stored on a secure server. Analysis will be conducted on these coded datasets to record medication changes and deprescribing.

### Confidentiality

Only É.B.-C. will have access to the nominal MedSafer data, which has the identity data encrypted and password protected at the level of the user account. MedSafer patient reports generated by the system must be nominal such that they can be given to the correct patient. These will be printed within the hospital, hand delivered, and stored in a secured area in the dialysis unit until needed. Following completion of the study, the printed MedSafer reports will be securely destroyed.

### Statistical Methods

#### Statistical methods for primary and secondary outcomes

Baseline demographics will be expressed as numbers and percentages for categorical variables and median (interquartile range [IQR]) for continuous variables. Differences between the intervention and control patients will be compared by χ^2^ or rank sum as appropriate.

For the primary outcome, we will use a mixed-effects logistic regression model controlling for the unit of intervention. As we expect some baseline differences in patient complexity between the control and intervention units whereby patients on the control unit tend to be more medically complex, we will adjust for the Charlson comorbidity index,^35^ median number of medications, and median number PIMs, as fixed effects. Adjusted risk differences will be estimated from the model parameter differences. A similar analysis will be conducted for the key secondary outcome. Analyses will be conducted in Stata Software Version 17 (StataCorp LP, Corpus Christi, USA).^36^

There are no planned *interim analyses*.

### Methods for Additional Analyses

A planned subgroup analysis will be conducted by biologic sex and by age category (<65 vs >65). The analytic approach will be the same as that used for the primary outcome analysis.

### Methods in Analysis to Handle Protocol Non-Adherence and Any Statistical Methods to Handle Missing Data

The study will be analyzed according to the intention to treat principle. Patients who are hospitalized, transplanted or who die prior to receiving a MedRec will be excluded from the final analysis. Missing data will not be imputed.

### Plans to Give Access to the Full Protocol, Participant-Level Data, and Statistical Code (31c)

The full protocol will be published online, and the anonymous participant-level data required to replicate the final study manuscript will be made available within 3 months of publication.

### Oversight and Monitoring

#### Composition of the data monitoring committee, its role, and reporting structure (5d and 21a)

Not applicable.

### Adverse Event Reporting and Harms

MedSafer reports have been extensively tested on older adults with polypharmacy in the acute care setting (including patients receiving maintenance hemodialysis).^16^ If a clinician suspects that a report contains an erroneous recommendation or that an adverse event has occurred secondary to the intervention, they will contact the study team to report it.

### Frequency and Plans for Auditing Trial Conduct

Not applicable.

### Plans for Communicating Important Protocol Amendments to Relevant Parties (eg, Trial Participants, Ethical Committees)

Not applicable.

### Dissemination Plans

Study results will be made available through publication in a peer-reviewed indexed journal. Study results will be shared at a leading annual conference on nephrology and/or general internal medicine.

## Discussion

This quality improvement study integrates electronic deprescribing decision support with the usual process of MedRec taking place on our dialysis units. To our knowledge, this is the first study to test the implementation of the new dialysis-specific deprescribing algorithms in a real-world setting. Potential implications of this quality improvement study include reducing the number of PIMs prescribed and decreasing pill burden by reducing the mean number of medications prescribed. This is a pragmatic study as it is built into the existing workflow for performing MedRec at our hospital center. As many dialysis units have similar MedRec procedures, we expect our findings would be scalable to other centers.

There are barriers to deprescribing during routine MedRec in hemodialysis. In fact, clinicians can lack sufficient time to complete a medication review and subsequently deprescribe; patient values and previous lived experiences with their medications, their perceived therapeutic effects, and potential adverse events are important factors to consider.^37^ Because of their chronic renal disease and complex medication regimens, patients on dialysis can also experience cognitive impairment that may affect the clinician’s ability to comprehensively perform medication reconciliation given already limited time constraints.^21^ Patients may also lack sufficient resources to learn about the medications they are taking, including their potential harms. The therapeutic relationship between the patient and the clinician can also influence initiation of a deprescribing trial or affect readiness to attempt a drug deprescription.^38,39^ This study aims to overcome these barriers through its methodology; we aim to address the lack of time by providing nephrologists with prepared deprescribing reports.

Whenever possible, patients should be involved in the deprescribing process. Patients may be reluctant to have certain drugs deprescribed because they have been taking them for a long time, and/or are wary of withdrawal or rebound side effects, such as acid reflux, inability or difficulty sleeping, or pain. This is a potential challenge that we hope our study methodology addresses by providing patients with deprescribing empowerment brochures for some relevant drug classes. The goal of the deprescribing brochures is to generate cognitive dissonance with an introductory quiz and increase motivation to deprescribe. We also aim to overcome the challenge of limited patient resources by providing patients with deprescribing brochures that use accessible language.

This study has several important limitations that need to be recognized. First, the study cohort is closed, so the sample size may, over the course of the study, decrease due to deaths, hospitalization, or transplant. We do not expect this to majorly affect the overall sample size as the duration of the intervention is short (taking place over 2 months). In this study, we do not currently have the resources to follow patients long term and are investigating the short-term impact of providing deprescribing reports on a single MedRec. The long-term impact on polypharmacy would need to be the subject of a further funded study. This study is also not powered to observe an effect on hard outcomes such as death, adverse drug events or hospitalization, especially in the short term. We will be reporting the number of gastrointestinal bleeds numerically, but we will not be powered to demonstrate a difference between intervention groups.^40^ This is a single-center study, and to influence outcomes such as these likely requires more than 6000 patients (based on our previous study of MedSafer in the acute care setting). This study is also not randomized; we aim to minimize (but cannot eliminate), through statistical adjustment, obvious baseline differences in comorbidities between the control and intervention units. Next, our study is not sufficiently powered to capture differences in rates of represcribing between the control and the intervention group. It should be noted that our study will capture prescribed/deprescribed drugs, and not *dispensed* drugs, or drugs actually taken by the patient. Another caveat is that our dialysis units do not have the support of a pharmacist, and so the MedRec process will be performed by the doctor and nurse on the unit. Finally, our study is taking place at an academic hospital center and so the patients may not resemble those treated in all dialysis centers. That said, the 2 units participating in the study tend to provide dialysis for general nephrology patients, and the intervention unit is attached to our community hospital site.

## Supplemental Material

sj-pdf-1-cjk-10.1177_20543581231165712 – Supplemental material for Electronic Decision Support for Deprescribing in Patients on Hemodialysis: Clinical Research Protocol for a Prospective, Controlled, Quality Improvement Study Click here for additional data file. Supplemental material, sj-pdf-1-cjk-10.1177_20543581231165712 for Electronic Decision Support for Deprescribing in Patients on Hemodialysis: Clinical Research Protocol for a Prospective, Controlled, Quality Improvement Study by Émilie Bortolussi-Courval, Tiina Podymow, Emilie Trinh, Joseph Moryousef, R. Hanula, Jean-François Huon, Thomas Mavrakanas, Rita Suri, Todd C. Lee and Emily Gibson McDonald in Canadian Journal of Kidney Health and Disease
