# Supplementary material for: Electronic Decision Support for Deprescribing in Patients on Hemodialysis: Clinical Research Protocol for a Prospective, Controlled, Quality Improvement Study
Source: Can J Kidney Health Dis. 2023 Jun 26;10:20543581231165712. doi: 10.1177/20543581231165712 (PMC10331104; doi:10.1177/20543581231165712)
Supplement: sj-pdf-1-cjk-10.1177_20543581231165712 – Supplemental material for Electronic Decision Support for Deprescribing in Patients on Hemodialysis: Clinical Research Protocol for a Prospective, Controlled, Quality Improvement Study [file sj-pdf-1-cjk-10.1177_20543581231165712.pdf]

General deprescribing fact sheet distributed to all hemodialysis patients on the intervention unit

Is it time to review your medications?

Canadian medication Appropriateness and Deprescribing Network

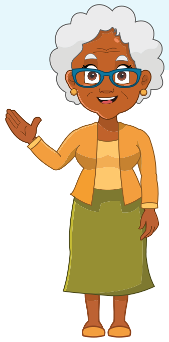

Medication use is a fine balance

Medications can help us in many different ways. But medications can also cause us harm. That's why it's important to weigh the potential benefits and harms of taking a medication over time.

What is **medication overload**?

Medication overload means taking more medications than we need. It also means taking too many medications that, together, cause more harm than good.

What are **too many medications**?

There is no strict number. When we take even one medication that can cause more harm than good at a particular time in our life, one can be too many.

Medication overload causes harm

Medication overload can cause drug interactions and harmful side effects. Harms from medication overload can be very serious. Some examples include:

- **falls & fractures**
- **hospitalizations**
- **premature loss of independence**
- **confusion & memory problems**
- **car crashes**
- **death**

Who is at highest risk?

People who take multiple medications, older adults, and women are at greatest risk of medication harm. The more medications we take, the greater our risk of experiencing harm.

1 in 10 **hospital admissions in older adults are the result of a medication side effect[1].**

What can you do? Deprescribing may be an option.

Deprescribing means working with your doctor or another health care professional to stop or reduce the dose of a medication that you feel may cause you harm or is not helping you.

Version 2.0: 2022/09/14

Flip the page for tips on preparing for a medication review. →

Preparing for a medication review with your doctor, pharmacist or nurse

Canadian Deprescribing Network

1. **Book an appointment** with your doctor, pharmacist or nurse *specifically* to review your medications.

2. **Questions to ask yourself before your appointment:**

- How are my medications affecting me? Am I having any problems with them?
- If my doctor recommended that I stop taking one or more of my medications, would I be willing?

3. **Prepare your list of questions in advance!**

Here are 5 questions to ask your doctor, pharmacist or nurse when starting a new medication or reviewing one you are already taking:

1. **Why am I taking this medication?**
2. **What are the potential benefits and harms of this medication?**
3. **Can it affect my memory or cause me to fall?**
4. **Can I stop or reduce the dose of this medication (i.e. deprescribing)?**
5. **Who do I follow up with and when?**

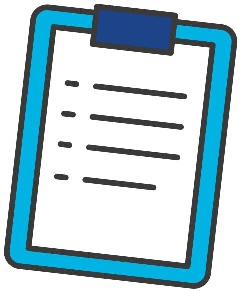

Remember to write down any other questions you would like to ask about your medications, too.

4. **Bring an up-to-date medication list to your appointment.** Ask your pharmacist for a list of all your medications, or make your own (**visit DeprescribingNetwork.ca for a sample record**). Include over-the-counter medicines and supplements.

Learn more about deprescribing and medication safety at DeprescribingNetwork.ca

References

1. Parameswaran Nair, N., Chalmers, L., Connolly, M., et al. (2016). Prediction of Hospitalization due to Adverse Drug Reactions in Elderly Community-Dwelling Patients (The PADR-EC Score). PLoS One, 11(10): e0165757. https://doi.org/10.1371/journal.pone.0165757

Statistical code in STATA[34] for the analyses

```
power twoproportions 0.1 0.25, test(chi2) n(214) nratio(0.5)
nfractional

Study parameters:

        alpha =    0.0500
            N =  214.0000
           N1 =  142.6667
           N2 =   71.3333
        N2/N1 =    0.5000
        delta =    0.1500  (difference)
           p1 =    0.1000
           p2 =    0.2500

Estimated power:

        power =    0.8023
```

How to complete a MedSafer MedRec – Lachine Campus – for Physicians

1. Gather the list of patients you will perform a MedRec on today
    a. We recommend doing 3-4 MedRecs per day, in a 5-day week on service.
    b. This week let's aim to do 17 MedRecs.
2. Identify the patients' MedSafer deprescribing reports in the binder called MEDSAFER REPORTS. The reports are sorted in alphabetical order, according to the patient's last name.
3. Review the MedSafer deprescribing report for one patient
    a. Review the deprescribing brochures attached for this patient and hand them to the patient
    b. Provide explanations on the topic of deprescribing and the benefits/risks of deprescription the proposed medication.
4. Document, in the NephroCare clinical note, the MedSafer MedRec completion, its date
5. In the clinical note, detail the changes implemented (to facilitate data collection for the study lead)
6. In NephroCare, adjust the MAR according to the MedRec changes 7. Document, on the MedRec sheet available on the clinical unit, that the "MedSafer MedRec done".
8. Once the MedSafer deprescribing report has been used, please attach it to the back of the MedRec sheet.
9. Repeat steps 3 to 8 for all patients you are doing MedRecs for.

If you have any questions, please contact the study lead, [redacted].
Email: [redacted]
Phone number: [redacted] (feel free to call/text this number)

The principal investigator of this study is [redacted] and [redacted] is the liaison between the research team and the hemodialysis team at the Lachine campus' outpatient hemodialysis clinic.

Below is the summary of the study we are doing

A Quality Improvement Deprescribing Intervention on the MUHC Dialysis Units

Revised Standards for Quality Improvement Reporting Excellence (SQUIRE 2.0) September 15th, 2015

Text section and item name	Section or item description
1. Title and abstract	Title
2. Abstract	Abstract
Introduction	Why did you start?
3. Problem description	Background and rationale
4. Available knowledge	Background and rationale
5. Rationale	Background and rationale
6. Specific aims	Objectives
Methods	What did you do?
7. Context	METHODS: Participants, Intervention and Outcomes (Study setting)
8. Interventions	Interventions (Explanation of the choice of comparators and Intervention description)
9. Study of the interventions	DATA COLLECTION AND METHODS
10. Measures	Plans for assessment and collection of outcomes<br><br>Data management
11. Analysis	Statistical methods
12. Ethical considerations	Recruitment
Results	What did you find?
13. Results	N/A
Discussion	What does this mean?
14. Summary	DISCUSSION
15. Interpretation	DISCUSSION
16. Limitations	DISCUSSION
17. Conclusions	DISCUSSION
Other information	
18. Funding	Funding
